# Effects of capture on acute and long-term reflex impairment, survival, and health of a deepwater fish: Shortspine thornyhead (*Sebastolobus alaskanus*)

**DOI:** 10.1371/journal.pone.0276132

**Published:** 2022-10-13

**Authors:** Cara Rodgveller, Christiane V. Löhr, John A. Dimond

**Affiliations:** 1 Auke Bay Laboratories, Alaska Fisheries Science Center, National Marine Fisheries Service, National Oceanic and Atmospheric Administration, Juneau, Alaska, United States of America; 2 Department of Biomedical Sciences, Carlson College of Veterinary Medicine, Oregon State University, Corvallis, OR, United States of America; University of Windsor, CANADA

## Abstract

Shortspine thornyhead (*Sebastolobus alaskanus*) are a benthic, deepwater species in the family *Scorpaenidae*. They have been tagged annually in Alaska since 1992, but have a low tag return rate of 1.6%. This may be at least partially attributed to post-release mortality related to capture. In this study, 21 shortspine thornyhead were caught on bottom hook-and-line longline gear and immediately given reflex tests. Eighteen were transported to the laboratory and held for 10–42 days, given reflex tests again, and then given postmortem examinations, including histopathology of tissues; three were given postmortem examinations after reflex tests on the vessel. There were no histological findings that could be directly linked to capture and holding; however, there were occurrences of myxozoan (protozoa) and metazoan (nematode) parasites, sometimes associated with minor inflammation. The vibration response reflex was found in only 24% of fish on deck and in 56% of fish after holding in the laboratory. The vestibular-ocular response was present in 47% of fish on deck and 89% of fish in the laboratory. A fish’s ability to right itself was successful on deck in 43% of fish (an additional 19% responded slowly) and 100% in the laboratory. Some reflex impairments may be permanent or may take more than days or weeks to improve. Reflex responses to other tests, the tail grab, gag, and operculum flare, were 95–100% successful on deck and later in the laboratory. A lack of reflexes may increase the risk of predation after release and may affect other behaviors related to survival and productivity.

## Introduction

Shortspine thornyhead (*Sebastolobus alaskanus*) (SST) are captured in fisheries along the continental slope at depths of 200–700 m and are tagged on annual National Marine Fisheries Service (NMFS) surveys in the Gulf of Alaska (GOA), Bering Sea, and the Aleutian Islands [1, 2]. SST are long-lived, slow growing, and the great majority of SST that are caught in fisheries range from ~20–60 cm [2, 3]. They can be retained, as SST have a high value per pound; however, the discard rate was 8–41% in the GOA from 2010–2020 [2]. There is a long standing tagging program that tagged 13,897 SST from 1992 to 2016 (500–1,000 each year) and 228 (1.6%) were recaptured during surveys or in bottom longline and trawl fisheries [4]. Fish have been recaptured after as few as 2 days at liberty or as long as 15.5 years. When returned with lengths and location these data can be used in analyses of growth and movement [4]. Other species tagged on the same surveys in Alaska have had higher recapture rates; the sablefish (*Anoplopoma fimbria*) tag return rate is 9% [5] and 7.4% of archival tags that were surgically implanted into Greenland turbot (*Reinhardtius hippoglossoides*) were returned [6]. Although in the same family as rockfishes (*Sebastidae*), SST do not have a swim bladder and, therefore, do not experience barotrauma during capture. They are expected to survive capture, tagging, and release as they do not have obvious injuries or behavioral impairments. The low recapture rate of SST may indicate that they suffer capture-related mortality; however, no studies have evaluated what stressors may negatively affect SST survival and health after capture.

SST is a federally managed species in Alaska, present mainly on the continental slope. The total allowable catch is apportioned to five management areas (Bering Sea, Aleutian Islands, Western GOA, Central GOA, and Eastern GOA) [2]. These management regions may not be sufficiently small enough to capture stock structure if dispersal distances are smaller than these management areas. The existing tag recovery data for SST demonstrates that movement among management areas is rare and indicates that this species is more likely to stay nearby their catch and release location than to move long distances [4]. With the limited tag and recapture data for SST, 73% moved <18.5 km [4]. However, there is concern that the small recovery sample sizes limit the ability to analyze movement rates and area-specific or stock-specific growth [4]. If management areas are too large, small regions within them could be overexploited, resulting in localized depletion. For example, catch and survey abundance of species in the same family, blackspotted (*Sebastes aleutianus*) and rougheye rockfish (*S*. *melanostictus*), decreased in some areas and not in a neighboring area, indicating that there may have been overexploitation and that the depleted areas may not be replenished by nearby, healthy populations [7]. Understanding stock structure is critical for a valid stock assessment and can be considered for spatial management. Regular evaluations of stock structure are required by the North Pacific Fishery Management Council. An increase of the amount of tag data and more information on how to interpret tag data, given potential health effects of capture and tagging, will provide a more informed analysis of dispersal rates and movement of SST across management boundaries.

Reflex impairment tests have been used extensively to develop models to predict acute and delayed mortality of fish after simulated trawling (e.g., [8–10]), fishery trawling (e.g., [11]), simulated hook and line angling [12], and sorting of fish in aquaculture [13]. Reflex impairments are a sign of stress, which can make fish susceptible to disease and parasites and can cause impaired growth, reproduction, predator evasion, and acute or delayed mortality [10, 14]. Tests include automatic responses to touch, vibration, visual cues, and the ability to self-orient (e.g., [14]). Observations of blood physiology, and sometimes injury, have been used in conjunction with reflex tests (e.g., [15, 16]); however, we have not identified studies that evaluated the effects of stress on tissues by histological evaluation, which can be used to identify parasites, microorganisms, inflammation, degeneration, necrosis, overall cellular health, type and severity of each, and aid in identification of the mechanism of acute or delayed mortality. If there is a correlation between on-deck reflexes or health, and subsequent acute or delayed health or mortality, reflex tests may be used as predictors of how fish will fare after capture and release. Collecting these data on deck could improve the interpretation of tag, release, and recovery data and the implications of releasing SST.

The objectives of this study were to evaluate 1) if capture from deep water affects the immediate reflex performance, 2) if reflex impairment is related to acute or delayed mortality, 3) if reflex impairment is related to long-term tissue health, and 4) if tissue health can be used to describe the mechanism of mortalities or reflex loss.

## Methods

### Ethics statement

The National Marine Fisheries Service Animal Care and Use Policy (04–112) does not include any requirements for research on captive or wild fish. Fish used in this study were collected and handled in accordance within the guidelines of the U.S. Government Principles for the Utilization and Care of Vertebrate Animals Used in Testing, Research, and Training (https://olaw.nih.gov/policies-laws/phs-policy.htm, accessed on 4/18/22) and the American Fisheries Society Guidelines for the Use of Fishes in Research (https://fisheries.org/docs/policy_useoffishes.pdf; Chapter V, accessed on 4/18/22). This work was completed under The Alaska Department of Fish and Game, United States permit CF-19-084. This permit allows the capture and transport of shortspine thornyhead to the National Marine Fisheries Service laboratory in Juneau, Alaska, United States and to be held on site. No species listed as threatened were captured. Reports on catch and disposition were delivered and reviewed for permit compliance by the Alaska Department of Fish and Game.

### Sampling

A commercial longline vessel, F/V *Seaview*, was chartered to conduct fishing operations between 57.37 N, -134.77 W to 57.73 N, -134.89 W in Lynn Canal, southeast Alaska from June 30 –July 1, 2019. Gear was set using 18/0 circle hooks spaced 2.7 m feet apart at depths from 437 to 658 m and was soaked for 3 hours to minimize the potential for copepod infestation (*Orchomenella* cf. *pacifica*). Immediately after capture SST were chosen for the study if they were not bleeding from the gills, which has been indicative of mortality in past surveys (unpublished data). Weights (g) and length (cm) were measured on deck and again in the laboratory after holding, prior to postmortem examination. The difference in length and weight were examined for indications of declining or positive health.

### Reflex tests

We conducted a series of tests on deck to determine if there was a lack of any reflexes immediately after capture. Fish exert effort while being brought up from depth and can also be injured during capture and release. The reflexes can be used as an indicator of future health or mortality. The same reflex tests were conducted in the laboratory prior to euthanasia. Fish were euthanized by rapidly severing the spinal cord behind the head.

Immediately after capture, fish were placed individually into a plastic container 90 cm long × 34 cm wide × 40 cm high filled with 60 l of seawater chilled to 3.5–4.0°C for performing reflex tests. The container was large enough so that movement was not restricted. SST had ample distance between them and the water’s surface, and the temperature remained stable over the 1 to 2 minutes it took to perform the tests. In this container we conducted a series of six reflex tests, which are reviewed in [9]. First, a gag reflex test was conducted by holding the head with one hand for stability and inserting a finger into the buccal cavity and touching the throat. A positive response was flaring of the operculum and opening of the mouth more widely [10, 17]. Second, an operculum flare reflex was tested by opening the mouth wide and releasing, with the expectation of a flare and then a return to an unflared, closed mouth [10, 17]. Third, the vestibular-ocular response was tested by holding the fish above the surface of the water with two hands with minimal pressure and rotating the fish 90 degrees to the right and the left around the long body axis. A positive reflex was when the eyes tracked the tester or another object [10, 17]. Fourth, a reflex to vibration was tested by tapping on the side of the holding tank and watching for a startled movement, sideways or forward [9]. Fifth, while the fish was sitting calmly, the tail was grabbed and released and a positive response was when the fish swam forward [18]. Sixth, the fish was turned upside-down and a positive reflex was recorded if it quickly righted itself [18]. A slow response to self-righting was also noted.

### Holding

After reflex tests, 18 SST were held in aerated, chilled water in insulated totes with lids for 1–2 days on the vessel. Water was maintained at 4.0–5.0 ⁰C. The fish were then transported to the National Oceanic and Atmospheric Administration, NMFS, Auke Bay Laboratories in Juneau, Alaska, where they were maintained in 24 hour darkness in filtered seawater chilled to 3.5–4.0°C. During the NMFS bottom hook-and-line surveys on the continental slope of the GOA, temperatures typically range from ~3.75 to 4.5°C between 300 and 800 m. Red light was used when feeding or conducting experiments in the laboratory as deepwater fish are sensitive to only blue light at wavelengths from 450–500 nm, encompassing the bioluminescent and residual daylight available below 200 m [19].

In the laboratory, fish were randomly divided into groups of seven and maintained in three, Frigid Units Inc. insulated Living Stream System® tanks, with their false bottoms removed and Styrofoam used as a lid to maintain the temperature. These rectangular, 530 l tanks have inflow and outflow ends; water was not recirculated and the flow rate was 34 l/hr. Water was chilled in a separate 2,271 l tank and was fed to the rectangular tanks through insulated pipes. After the first week, fish were fed twice a week until satiation over the course of 30 minutes.

Fish were held for increasingly long periods before being euthanized to identify possible delayed effects on health or reflex test outcomes. Fish were either tested on deck (3 fish), after 10–11 days (4 fish), 17–19 days (4 fish), 28 days (5 fish), or 42 days (5 fish) after capture. At each point fish were tested for each reflex, length and weight were measured, fish were euthanized, postmortem examinations were performed, and tissues were collected for a histological evaluation, as described below.

### Postmortem examination and histology

After the experimental treatment and euthanasia, both eyes were examined to highlight any abrasions, lesions, or parasites on the cornea by applying a solution of fluorescein (100 mg/l) and sodium bicarbonate (800 mg/l), used to neutralize the pH of the fluorescein. Fluorescein is commonly used on human eyes and fish skin to detect corneal or cutaneous damage [20]. The solution pools into or adheres to irregular and damaged surfaces and is visible in darkness when illuminated with ultraviolet light (480–600 nm) [21]. Eyes were also examined for cloudiness or other aberrations.

After fish were euthanized, a postmortem examination (necropsy/autopsy) was conducted. Tissues were collected for histological evaluations, including the gills, skin, eyes, brain, heart, liver, spleen, intestines, stomach, gonads, kidney, muscle, and, in a few cases, the vent and pharynx. Tissues were immediately placed in Excell Plus Fixative ™ and stored at room temperature. After completion of the experimental portion of the study, samples were shipped to the Oregon Veterinary Diagnostic Laboratory (OVDL) at Oregon State University for further processing.

Tissues were processed to paraffin-embedded blocks following standard operating procedures at the OVDL. In brief, after trimming tissues to fit cassettes, tissues were processed overnight to replace water with increasing concentrations of ethanol, followed by xylene, and ultimately paraffin. From each paraffin block, a 3–5 μm section was cut, deparaffinized, stained with hematoxylin and eosin (H&E), dehydrated, and cover slipped. Slides were examined by bright-field microscopy by a board certified veterinary anatomic pathologist (CL) using a Nikon Eclipse E400 microscope. Each tissue was examined histologically for presence of any findings outside the normal limits including but not limited to inflammation (cellular infiltrates, exudate, granulations tissue, fibrosis), degeneration, necrosis, cellular proliferation, and presence of internal or external infections by organisms such as parasites and microorganisms (including bacteria and fungi) [22, 23].

## Results

### Conditions

Twenty-one fish were captured in total: three fish were euthanized and sampled on deck and 18 fish were transported to the wet laboratory. All 18 laboratory fish survived culture and experiments. Air temperature on deck was approximately 14.5–16.5 ⁰C and temperature in the laboratory was approximately 13.0 ⁰C, as it was cooled by the abundant, chilled water. Fish were exposed to air for approximately 15 seconds two times while on deck.

### Growth

The mean body length on deck was 47 cm, with a range of 37–56 cm. On average, for all fish held in the laboratory, from capture date to the date of sacrifice, length increased by an average of 0.4 cm over the course of the study and weight increased by 0.2 kg. SST are slow growing and so we did not expect to see growth, except potentially over the longest time periods if fish did not feed. The largest average increase in length was at time period 3 (0.8 cm) (28 days) and there was a decrease in length in time period 4 (-0.2 cm) (42 days). The fluctuations in length indicate that the difference may be due to low sample sizes and measurement error. The largest increase in weight was at time period 2 (0.5 kg) (17–19 days) and the lowest increase was at time periods 3 (0.03 kg) and period 4 (0.08 kg), i.e., the weight was the same as capture at time periods 3 and 4.

### Reflexes

**Fig 1:**
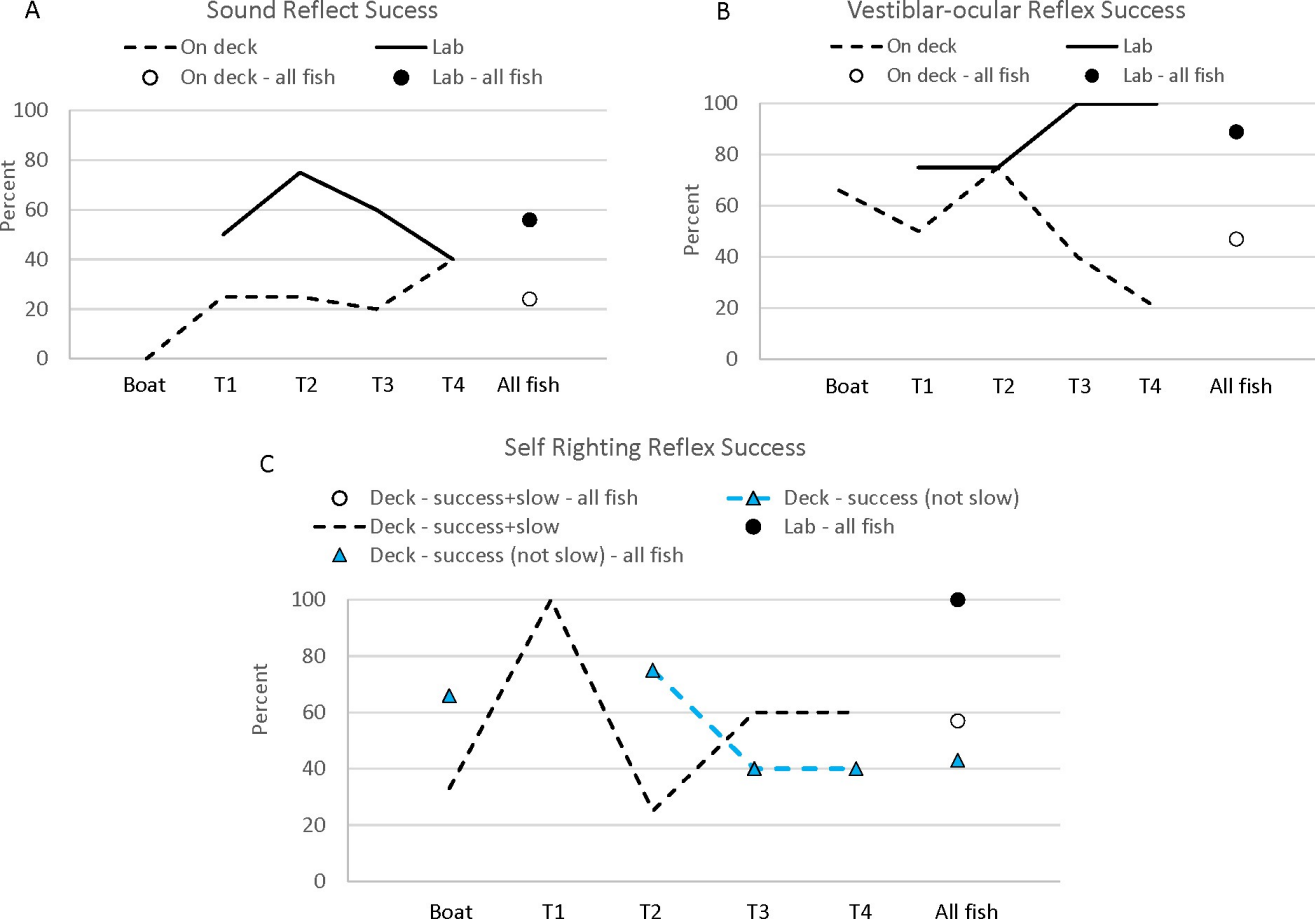
The percent of shortspine thornyhead that successfully performed each reflex test, or performed them slowly (for self-righting), on deck and 10–42 days later in the laboratory. Fish were tested in the laboratory (“Lab”) or immediately after capture (“On deck”). “On deck” reflex tests are presented for fish euthanized at each time period (T), where the reflex result is “On-deck” and not at the respective time period. Holding time periods included: 10–11 days after capture (T1), 17–19 days (T2), 28 days (T3), or 42 days (T4). The sample size of fish tested for reflexes and subsequently euthanized were: boat = 3, T1 = 4, T2 = 4, T3 = 5, T5 = 5. Overall, there were 21 fish tested on deck (“On deck—all fish”), which included the18 fish transported and held in the laboratory (“Lab–all fish”).

Reflexes were tested on deck (N = 21) and then again after holding in the laboratory (N = 18). Shortspine thornyhead had a reaction to the tail grab (100%), gag (95% on-deck and 100% in the laboratory), and operculum flare reflex tests (95% on deck and 100% in the laboratory). On deck, 43% of SST were immediately successful in righting themselves on deck and 19% were slow and eventually successful; in the lab, 100% of the fish succeeded rapidly (Fig 1).

Fish were often not successful at responding to vibration; 24% of fish responded to vibration on deck and 56% in the laboratory (Fig 1). The vibration response in the laboratory was poorest for the group held for the shortest number of days and the longest number of days (10–11 days 50%; 17–19 days 75%; 28 days 60%; 42 days 40%). This demonstrates that the time in the laboratory was not related to improvement in the vibration response. When examining the changes in individuals, at 10–11 days and at 17–19 days 50% of fish improved and 25% lost the reflex; at 28 days 40% improved and 40% lost the reflex; and at 42 days 20% improved and 20% lost the reflex. This indicated that although the overall trend was to redevelop the vibration reflex after holding, some fish lost the reflex while in the laboratory as well.

The vestibular-ocular reflex test, where fish eyes tracked the observer, had an 89% success rate in the laboratory and only 47% on-deck. In the latest two holding time periods in the laboratory, 100% of the SST had a vestibular-ocular reflex and in the first two time periods 75% of fish had the reflex (Fig 1). Only one fish lost the reflex after having one on the boat; this was at 17–19 days. This result shows that most SST recover the vestibular-ocular reflex and the proportion of fish may increase with time. In summary, capture affects some reflexes acutely and some of these reflexes improve with time for some fish, while other impairments may be permanent or take more than 42 days to recover.

### Histology

We examined both eyes with a fluorescein test, a visual examination, and histologically. These tests were conducted to highlight any abrasions, lesions, infections, barotrauma, or parasites that may have been naturally occurring, caused by acute trauma, or developed or worsened after holding. There was no cloudiness or any other lesions seen in the cornea macroscopically with or without fluorescein after capture or after holding. There were no parasites and no histologic changes identified in ocular tissues including in the cornea, anterior chamber, lens, posterior chamber, retina, and sclera.

Two different issues were present in some of the examined hearts (Tables 1 and 2); endocardial hypertrophy, the enlargement of cells lining the inner surface of the heart, was documented in a single fish (5%), which was sampled on deck (Tables 1 and 2). Endocardial hypertrophy is suggestive of immune stimulation without a specific cause. Granuloma(s), a type of chronic inflammation that is the stereotypical response to any persistent inflammatory stimulus, was seen in the heart tissue of 7 out of 21 fish (33%). This inflammation was mild and infrequent and thus not likely to be associated with current or future health effects.

**Table 1:** Histological findings in shortspine thornyhead tissues.

Tissue	Abnormality	Description
Heart/ventricle (a)	Endocardial hypertrophy	Inflammation
Heart/ventricle (b)	Minimal granuloma; likely secondary to migrating parasites	Inflammation
Stomach (submucosa)	Granulomas with metazoan parasites (nematode)	Parasite; inflammation
Stomach/pyloric ceca epithelium (a)	Myxozoan parasite (protozoa)	Parasites with minimal or no tissue reaction
Stomach/pyloric ceca epithelium (b)	Nematode eggs, larvae, adults	Parasite with minimal or no inflammation
Intestine epithelium	Sparse protozoa	Parasites with minimal or no tissue reaction
Intestine (adventitia/mesentery)	Nematodes	Parasites with minimal or no tissue reaction
Liver (a)	Sparse nematodes	Parasites with minimal or no tissue reaction
Liver (b)	Sparse granuloma	Inflammation
Kidney (tubules)	Protozoa	Parasite; low grade infection with parasite; no tissue response
Skin	Minimal necrosis	Suspect stress response
Ureter epithelium	Protozoa	Parasite; low grade infection; no tissue response

**Table 2:** The percent of histological findings in tissues of shortspine thornyhead by treatment type.

Tissue	On deck (3)	T1 (4)	T2 (4)	T3 (5)	T4 (5)	Overall
Heart/ventricle (a)	33					5
Heart/ventricle (b)		50	25	60	20	33
Stomach submucosa	33	50		20	20	24
Stomach/pyloric ceca (a)				60		14
Stomach/pyloric ceca (b)	100	100	75	60	80	81
Intestine epithelium	33					5
Intestine/mesentery	33		25			10
Liver (a)		25		20		10
Liver (b)		25			20	10
Kidney tubules				20		5
Skin		25		20		10
Ureter epithelium	100	50		60	40	48

Fish were either sampled on deck, in the laboratory during time period 1 (T1), which was within 10–11 days after capture, 17–19 days (T2), 28 days (T3), or 42 days (T4). The number of fish in each treatment type are in parentheses in row 1. “Overall” is the percent of all fish represented in that column. There were 21 fish tested on deck and 18 in the laboratory (T1-T4); three fish were euthanized on deck.

**Fig 2:**
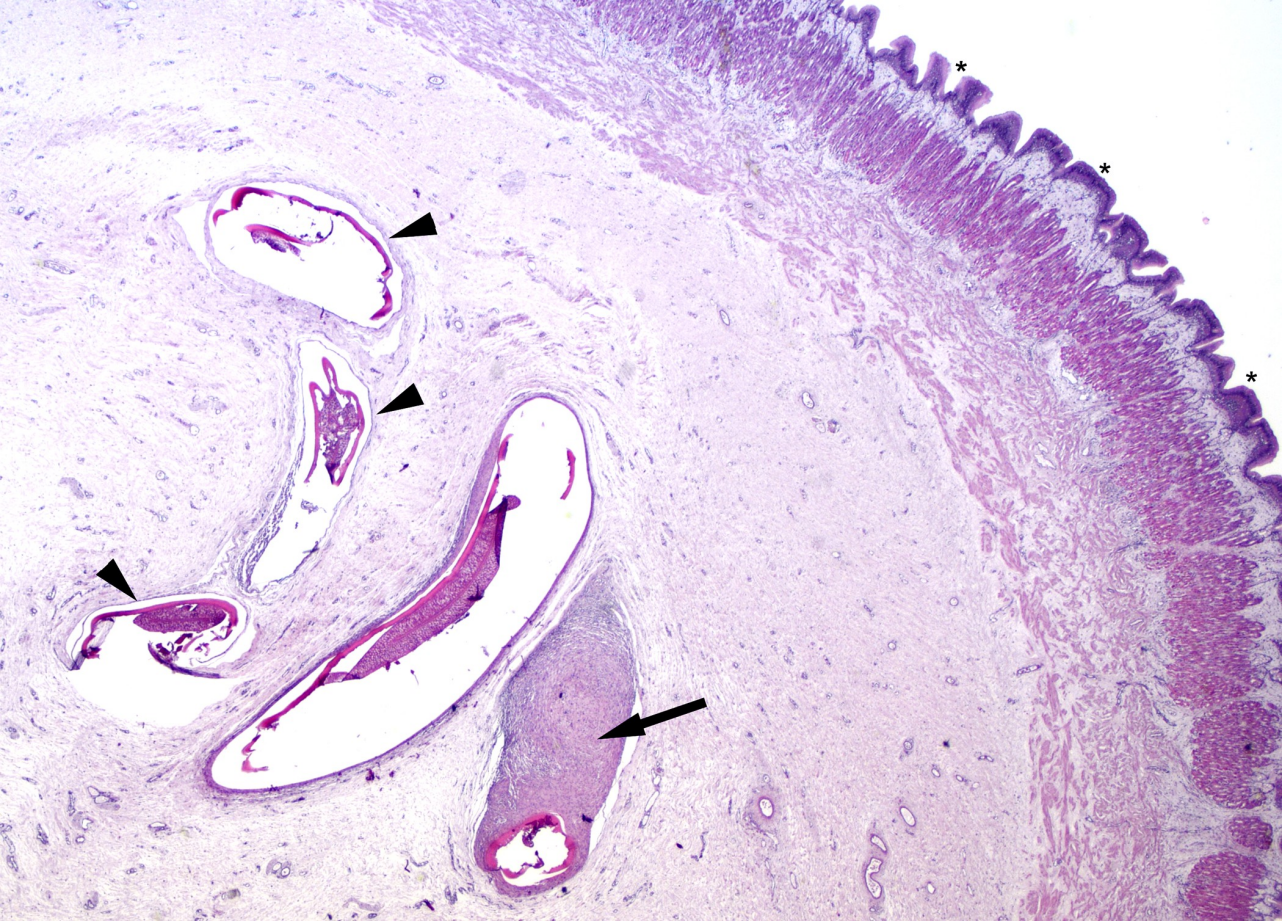
Cross section of a shortspine thornyhead (*Sebastolobus alaskanus*) stomach with multiple nematodes in the stomach submucosa. Asterisks are placed along the surface of the stomach mucosa. Nematodes are surrounded by negligible (arrow heads) to intense (arrow) granulomatous inflammation, which is the typical host response to parasites in fish. Magnified 40x; stained with hematoxylin and counterstained with eosin.

The digestive tract had evidence of parasites in the stomach including pyloric ceca and the intestine (Table 1). Nematode and protozoan parasites were identified in the lining (mucosa) of the stomach and pyloric ceca (Fig 2). In this context protozoa are myxozoan and metazoans were identified as nematodes when well preserved. The submucosa, the layer between the lining and the muscle layer of the stomach, had granulomas, some with nematodes, occasionally large enough to be visible to the naked eye. Nematodes were present in 5 fish (24%) across all treatment groups, except those sampled after 19 days (time period 2). In 17 fish protozoa were present in the submucosa location with no or minimal inflammation (81%). The stomach and pyloric ceca of 3 fish (14%), all sampled after 28 days, had non-granulomatous inflammation (granuloma are chronic), which may have been secondary to a protozoan parasite infection (Table 2). All findings in the stomach and pyloric ceca were interpreted as pre-existing conditions or background lesions. Regarding intestines, one fish (5%) had sparse protozoa in the wall of the vent; 2 (10%) had a few nematodes with minimal or no associated inflammation (Table 2).

**Fig 3:**
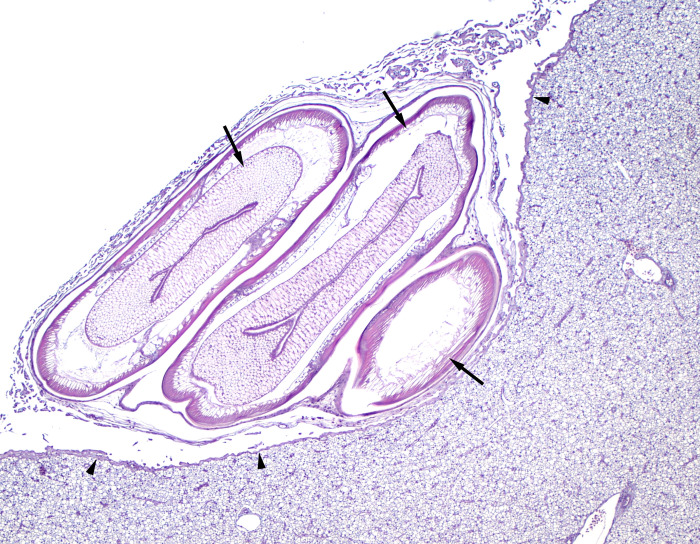
Cross section of a shortspine thornyhead (*Sebastolobus alaskanus*) liver and mesentry with multiple nematodes. Multiple nematodes (arrows) expand the mesentery adjacent to the liver (capsular surface of liver labeled by arrow heads). Magnified 40x; stained with hematoxylin and counterstained with eosin.

In the liver, kidney, and skin there were minimal findings; however, nematodes were present in the ureter of nearly half of the fish (Table 2). There were a few nematodes in the liver of 2 fish (10%) and infrequent granulomas without associated parasites in 2 fish (10%) (Table 2, Fig 3). The kidney of 1 fish (5%) had small numbers of protozoa in renal tubules without identifiable tissue response (Table 2). Two fish (10%) had minimal skin lesions in form of epithelial necrosis, one at 17–19 days and one after 28 days, which were not of concern at time of examination as mucus and superficial epidermal layers were intact (Tables 1 and 2). The tissue irritation may have be caused by mechanical or other irritation associated with capture or holding. Necrosis of epithelial cells in the skin can be seen with stress and, if severe, lead to clinically relevant lesions such as ulceration or fin rot.

The ureter epithelium had protozoan parasites in 48% of fish (Table 2). These were identified in all 3 fish sampled on deck and were present at laboratory sample time periods 1 (1–11 days post capture), 3 (28 days), and 4 (42 days) in the laboratory. This was the second most prevalent issue documented; like other histological observations, it was not caused by the experiments and in general is not likely to impact overall health and survival.

None of the findings in the tissues could be attributed to trauma or the experiments and none of the findings by themselves would be considered potentially fatal. There were histological findings in all time periods, with time period 2 (17–19 days post capture) having the least. Laboratory time periods 1 and 3 had the highest diversity in the types of findings and time period 3 had the most findings. There is no evidence that being in the laboratory caused an increase in histological findings. The absence of prominent inflammatory lesions associated with the organisms is consistent with adaptation or co-evolution of a host and parasite and low impact of parasites on overall health and survival [22, 23]. The only findings that showed up later in the experiment were the protozoal infections in the stomach/pyloric ceca in period 3 (3 fish) and 1 fish with nematodes in the kidney tubules.

## Discussion

There were no mortalities after capture on hook-and-line gear in deep water or after holding in a laboratory environment for 10–42 days. The histological tissue findings were minor; no clear associations could be established with capture or holding time, and many if not all were likely present prior to capture. Importantly, none of these histologic findings appeared to affect long-term health while in the laboratory and are highly unlikely to affect overall health in the future. However, there was an acute effect of capture on the reflexes relevant to orientation in the environment: self-righting, vibration, and vestibular-ocular reflexes. With long-term holding, the self-righting reflex returned in all fish. The absence of orientation and startle reflexes immediately after capture, orientation (43% immediate success; 19% slow), visual (47% success), and vibration reflexes (24% success), could all affect predation rate after release and on the descent to their benthic habitat at 200–700 m [1, 2]. While behavior may not reflect delayed post-release mortality immediately, if fish are tagged and released on deck, these negative reflexes may affect their survival in the wild, as automatic responses are integral to predator evasion and food searching [24]. Besides concerns over the mortality of tagged fish, SST are commonly discarded in fisheries (8–41% from 2010–2020) and there are no studies on SST post-release mortality [2].

The vibration response, which was absent in some fish throughout the study, may be important for released fish if they must evade predators that make vibration, termed acoustics when vibrations can be heard [14]. Like SST, the vibration reflex of sablefish (*Anoplopoma fimbria*), caught on the same hauls as the current study, was also absent in 43% of fish and it was absent from more fish when additional stressors were added experimentally [25]. Sperm whales (*Physeter microcephalus*) follow longline fishing vessels and the NMFS longline survey vessels in the GOA and use echolocation at frequencies from 100 Hz to 30 kHz [26, 27] to depredate fishing gear, when locating fish after they are removed from the hook (and before they can return to the benthos), and likely when identifying prey under natural conditions [5, 28–30]. Sablefish create vibrations from 344 Hz to 34 kHz [31], which are very similar to sperm whales. Although there is no evidence of SST creating vibrations, the sablefish results demonstrate that a species without a swim bladder may be able to create and detect cetacean acoustics at these frequencies. Sperm whales are natural predators of SST and so the vibration reflex may be important for more than the short period after release (estimated 24% of all SST predation mortality) [2].

Although SST lack a swim bladder the vibration reflex test may be a measure of the fish’s responsiveness to vibration in multiple ways. Fish are known to use otoliths and the lateral line to detect vibration pressure and particle motion [32–34]. These pathways would likely be sensitive to the vibration that was applied in this study because it was in close proximity to the fish. Although it may not be possible to isolate the cause of the lack of reflex in SST or understand the importance of any physical adaptations to detect vibration, it is important to note that there are multiple ways that fish may detect vibration. There are very few studies of deepwater fish vibration detection and vibration creation because of the logistics of working with deepwater species and vibration *in situ* and in the laboratory.

Benthic and pelagic species, or different intraspecific life stages, may not have equal necessity for each reflex. For example, SST are sedentary and are often resting on the bottom, either in depressions or in associations with bottom structure [3]. The reflexes needed by SST may be dissimilar to fish with different life histories, such as those living in the pelagic zone or schooling, or for species that are known to make vibration, possibly for communication, like deepwater sablefish [31]. It is unknown how a lack of these reflexes will affect a sedentary species if they are permanent. It is difficult to interpret the vibration response results because of the differences among individuals; whereas there was a clear improvement trend for other reflexes. It may be that the vibration test would need to be delivered in a more precise way or with varying frequencies.

We did not identify the thresholds of stressors that would cause reflex absence and subsequent acute or delayed mortality. To develop a model to predict mortality, for a given species and life stage, a gradient of a single stressor, like time out of water [25], or a suite of stressors, such as fishing [9, 18], would need to be applied. For example, simulated trawling affected the reflexes and mortality of five north Pacific ocean fish species differently (walleye pollock, *Gadus chalcogrammus*, sablefish, Pacific halibut, *Hippoglossus stenolepis*, and coho salmon, *Oncorhynchus kisutch*, and northern rock sole, *Lepidopsetta polyxystra*) [10, 18]. The severity of the stressor, in this case trawling, affected the reflexes of each species differently and required species-specific models for predicting mortality from reflexes. The point at which the stressor caused acute or delayed mortality in a portion of the fish must be identified for each species to develop individual predictive models.

SST live in deep, low-light waters and have proportionally large eyes. They likely utilize vision for detection of predators and prey, as they live in depths where there is still daylight: the mesopelagic zone [19]. In our study fish were exposed to light when they were captured and during reflex tests on deck and in the laboratory. It is possible that light exposure may damage parts of the retina, as SST are accustomed to low light environments. We did not see evidence of lasting retinal damage in any fish, as the vestibular-ocular reflex was successful in more fish after holding and there was no evidence of retinal atrophy on histologic examination. In controlled experiments, Pacific halibut (*Hippoglossus stenolepis*) were exposed to light and had reduced retinal activity over the 10-week experiment, indicating that there was long-term physical damage [35]. Despite the damage, their behavioral response to prey was not impaired in a low-light setting. The authors speculated that there may be other behaviors that should be tested to see if there are implications of retinal damage. For SST, it would be valuable to identify other behavioral tests that measure the practical implications of visual impairments, such as predator avoidance or feeding.

We did not test the effects of behavior impairment when fish are exposed to risks to health and survival in the natural environment. It would be important to test how long it takes for reflexes to improve in some fish. On the NMFS longline survey, it is possible to keep fish in a tank with refreshed seawater for observation. Testing reflexes hourly over the day may be used to identify a holding time that is practical for operations and for minimizing mortality after release due to a lack of reflexes. However, even if fish are held before release, there are still concerns over the implications of reflex issues that we saw in the laboratory after holding. There are also stressors that fish will be exposed to when in a holding tank on deck that we did not evaluate. These stressors, such as vibration/sound, warmer surface water, and light, may cause a loss of reflexes or acute or delayed mortality and should be evaluated in experimental studies if holding in tanks is considered as a method of recovering reflexes.

There were a few tissues with histologic findings (histopathology), even though fish appeared to be healthy and all survived. The great majority of findings were parasites, only sometimes accompanied by inflammation, with the most common being nematodes in the stomach/pyloric ceca and protozoans in the ureter. Issues were most common at time periods 1 and 3, and so there was no trend with time in captivity. Therefore, it is not likely that the inflammation and presence of parasites were caused by the experiment but rather were present prior to capture (background lesions). Because parasites were present throughout the study, it is likely that they are common in wild fish and do not pose a threat to overall health. The absence of prominent inflammatory lesions associated with the parasites further supports this notion. This is consistent with adaptation or co-evolution of a host and parasite [22, 23]; thus, the impact on overall health should be negligible after release into the wild.

Although we did not have any significant findings from the histological evaluation, unlike reflexes or blood physiology, histology can uniquely identify mechanisms behind delayed mortality. A lack of significant histological findings does not diminish the importance of in-depth evaluations of fish health, including histological evaluations of tissues. Pairing histological observations with reflex testing and observations of mortality can provide a full picture of the mechanisms behind mortality. Identification of lesions such as ruptures, inflammation, degeneration, necrosis, cellular proliferation, and disease can be used with behavioral responses to predict mortality [14, 22]. These evaluations are recommended for all experiments and observational studies, particularly when working with new stressors or a new species. It is necessary to work with a histopathologist who has worked with fish and can recognize which issues are commonplace or caused by stress, their severity, and what the related risks are to health. In any species, a comprehensive evaluation of the naturally occurring irregularities are advised, to serve as a baseline for comparison to fish tissues from experiments.

In the future, we recommend that more potential stressors that occur during capture, tagging, and release be tested on deck and experimentally in the laboratory. In the current study, we were able to look at the effects of the stress of capture on hook-and-line longline gear through being unhooked. It will be important to observe fishing and to identify a suite of stressors that fish are exposed to during the entire fishing operation. Each of these stressors should be tested individually and in combination to identify which factors are the most impactful to behavior, health, and mortality. If particular stressors are determined to be impactful, they may be minimized during fishing and tagging operations. *In situ* studies of survival after release would also be an important addition, and there are attempts to evaluate this with a large scale tagging program; however, SST recaptures are sparse and these studies are not an efficient way to measure mortality rates. Because we did not observe mortality, it is possible that the low recapture rates are related to a lack of a directed fishery [2, 4]. Without tagging studies, experimental work becomes even more critical.

In summary, SST survived capture and holding for up to 42 days. Fish sampled on deck and at time intervals from 10–42 days after capture did not show evidence of health concerns that would by themselves lead to mortality. However, due to the lack of visual, swim, and vibration reflexes, some fish may be at risk of immediate predation after release or delayed mortality due to issues with feeding or movement, for example. Some reflexes did improve with time; however, it is unknown how quickly some reflexes can return. The lack of mortalities in our study demonstrates that capture on longline gear may not directly cause acute or delayed mortalities; however, mortality after release in the wild is still unknown. Future studies should focus on the time it takes for reflexes to improve, the severity or time exposed to a stressor that will lead to mortality, and which specific stressors are incurred during fishing operations that may cause injury or impairment.

## Supporting information

S1 Data Data set underlying the shortspine thornyhead reflex study results. (XLSX)
